# A Randomized Controlled Trial to Test the Effects of Repetitive Peripheral Magnetic Stimulation Versus Neuromuscular Electrical Stimulation in Patients with Spastic Hemiparesis After Stroke (REPMAST): Study Protocol

**DOI:** 10.3390/brainsci14121249

**Published:** 2024-12-12

**Authors:** Kristin Loreen Pohl, Jens Müller, Katja Wittig-Böttger, Alexander Ritter, Farsin Hamzei

**Affiliations:** 1 Section of Neurological Rehabilitation, Clinic of Neurology, Jena University Hospital, 07747 Jena, Germany; kristin.pohl@moritz-klinik.de (K.L.P.);; 2 Department of Neurology, Gräfliche Kliniken Moritz Klinik GmbH, 07639 Bad Klosterlausnitz, Germany

**Keywords:** peripheral magnetic stimulation, stroke, rehabilitation, neuromuscular electrical stimulation, spasticity

## Abstract

Background/Objectives: Innovative therapies are needed to reduce disability, facilitate activities of daily living, and improve the quality of life in patients with stroke. Non-invasive methods of stimulating the peripheral and central nervous system are increasingly being used to enhance the effects of existing therapies in stroke rehabilitation. One potentially relevant method for achieving greater improvement is repetitive peripheral magnetic stimulation (rPMS). This randomized controlled trial (RCT), the Peripheral MAgnetic stimulation in patients with spastic hemiparesis after Stroke Trial (REPMAST), will investigate whether rPMS improves upper extremity function, spasticity, and activities of daily living in patients with stroke compared with neuromuscular stimulation (NMS). Methods: REPMAST is an interventional, randomized controlled single-blinded study. Patients with subacute stroke are randomized to receive rPMS or NMS five days a week for three weeks in addition to standard rehabilitation therapy. The primary outcome is the change in the Fugl–Meyer Assessment for Upper Extremity between the beginning and end of the stimulation sessions. Secondary outcomes include changes in the Katz Index of Independence in Activities of Daily Living, the Timed Up and Go Test, the Modified Ashworth Scale, and the Tardieu Scale. A total sample size of 138 patients (69 in each group) is required to investigate the superiority of rPMS compared with NMS. Conclusions: The aim of this RCT is to provide evidence for an effective peripheral stimulation treatment for stroke recovery.

## 1. Introduction

Stroke is the third leading cause of death and disability combined worldwide [1]. Innovative therapies are needed to reduce disability, facilitate activities of daily living (ADL), and improve quality of life [2].

In recent years, in addition to physiotherapy and occupational therapy, non-invasive brain stimulation methods such as transcranial direct current stimulation and repetitive transcranial magnetic stimulation (rTMS) have been increasingly used to modulate brain function in order to improve functional deficits after stroke.

Another possibility to modulate brain function is through peripheral application, such as peripheral electrical stimulation [3,4] or with the use of repetitive peripheral magnetic stimulation (rPMS) [5,6]. Repetitive peripheral magnetic stimulation (rPMS) is a painless stimulation method that uses rapidly changing magnetic fields to stimulate peripheral nerves and trigger repeated contractions of the skeletal muscles. Using rPMS is simple compared with other NIBS approaches. The stimulation coil is placed directly on the skin in the area of the target muscle to be stimulated. The magnetic field penetrates the tissue and causes depolarization due to the electric field that builds up, resulting in the induction of an action potential, which clinically leads to muscle contraction. The rPMS method has also been used to stimulate peripheral nerves or spinal nerve roots, with the stimulation coil placed paravertebrally or over the corresponding nerve [7,8,9,10]. The exact mechanism of how rPMS interacts with the central and peripheral nervous systems is not yet fully understood. Struppler and colleagues suggested that rPMS causes increased proprioceptive input to the brain by activating mechanoreceptors of the contracted muscle [11]. Another type of input has been suggested to come directly from the nerve fibers. Consequently, rPMS increases sensory input from the affected limb to the brain, initiating neuroplastic processes and leading to improved sensorimotor performance in patients.

In the 1990s, the first studies by Struppler and colleagues reported improvements in perception, spasticity, and paresis after stroke and in patients with multiple sclerosis following the use of rPMS [12,13,14]. Over the past decade, the number of studies using rPMS has increased significantly [15]. Recent meta-analyses have reported that rPMS induces a better Fugl–Meyer Assessment for Upper Extremity (FMAUE) compared with control groups [16,17]. However, it has also been criticized that there is a lack of rPMS studies conducted as RCTs with large sample sizes [18,19,20]. Therefore, the current randomized controlled trial, the Repetitive Peripheral Magnetic stimulation in patients with spastic hemiparesis after Stroke Trial (REPMAST), will investigate the effect of rPMS compared to neuromuscular stimulation (NMS) in a large sample of patients with stroke. Because of the sensory influence of rPMS on the brain, the effect of rPMS in REPMAST will be compared with that of a control group that receives neuromuscular stimulation (NMS).

Previous reports have compared the advantages and disadvantages of rPMS and NMS [5,6,21]. It is important to note that although both interventions stimulate some common peripheral structures [6], they nevertheless activate different networks. Repetitive PMS has been found to increase activation of the ipsilesional superior posterior parietal and premotor cortex [11]. By contrast, NMS has been described to increase activity in the ipsilesional sensorimotor cortex [22]. Recent clinical studies have compared rPMS over muscles with sham [23], standard care [21], conventional physiotherapy [24], or its combination with low-frequency rTMS [10]. REPMAST is a randomized controlled comparative interventional trial that aims to investigate the efficacy (superiority) of rPMS compared with NMS in a large number of patients with subacute stroke, as there is no direct comparison between rPMS and NMS.

There are conflicting results regarding the effectiveness of rPMS in improving spasticity [14,16,18,19,20,23]. Therefore, this study will also analyze the effect of rPMS on spasticity.

## 2. Materials and Methods

### 2.1. Aim of the Study

The aim of the study is to compare the efficacy of rPMS and NMS in patients with an arm paresis in the subacute phase of stroke.

This research will be conducted in accordance with the Declaration of Helsinki and was approved by the local Ethics Committee on 22 August 2014 (No. 4147-07/14). The study is registered in the German Clinical Trials Register under the number DRKS00007899 (Universal Trail Number: 1111-1301-9734). Written informed consent to participate in the study will be obtained from each patient prior to the start of the study. A copy of the informed consent form will be given to each enrolled patient.

### 2.2. Patient Population and Eligibility Criteria

Participation in the study is voluntary. Each patient has the right to withdraw from the study at any time without giving a reason (withdrawal of consent) and without suffering any disadvantage. The patient will be asked to state the reason for withdrawal but will be advised that he/she is not required to do so.

The inclusion criteria are stroke onset within the previous six months (defined as ‘subacute stroke’) and a relevant arm paresis with at least one point within the subscore C of the FMAUE and an MAS score of ≥1.

The exclusion criteria for this study include a history of recurrent stroke, abnormal brain findings on CT or MRI images, pacemakers or other implanted devices, metals in the affected upper extremity, a history of epilepsy in the patient’s medical history, neurodegenerative disorders, psychiatric disorders, pregnancy, the use of antispastic medication, and botulinum toxin A injections in the affected upper extremity within the previous six months. Patients will also be excluded if they have a language comprehension disorder that makes it difficult for them to understand the rationale of the study. Patients will be recruited from an inpatient neurorehabilitation clinic. All admitted patients will be screened for eligibility for the study. All eligible patients will be informed about the contents of the REPMAST. They will be enrolled when they sign the written informed consent.

### 2.3. Sample Size Calculation

REPMAST is designed to show the superiority of rPMS over NMS. Recently, the effect size of rPMS was calculated to be r = 0.68 for the FMAUE and r = 0.60 for the Wolf Motor Function Test when comparing rPMS with standard therapy [21]. A smaller effect size is expected here, as the comparison between rPMS and NMS contrasts two interventions. As there is still no RCT directly comparing rPMS and NMS, the final sample size was first compared by conducting a feasibility pilot study with 20 patients with stroke (10 in each intervention group) using the same inclusion and exclusion criteria as described above. According to the results of the feasibility pilot study, we found an effect size of 0.6 on FMAUE_Diff_ (the difference between Fugl–Meyer Assessment scores before and after the three-week stimulation session) for the rPMS group. Accordingly, to achieve a 90% statistical power to detect differences in FMAUE_Diff_ between the two groups at the 5% significance level (2-sided), a total sample size of 120 patients is required [25]. With an estimated drop-out rate of 15%, a total sample size of 138 patients (69 in each group) is required for this study.

### 2.4. Clinical Assessment

Demographic data will be recorded at the measurement time point ‘pre’ before the intervention. Imaging data are used to determine the territory affected by the stroke. This will be followed by a medical history and a physical and neurological examination.

The following scales will be used to quantify neurological deficits, as assessed by trained raters before the intervention (‘pre’), at the end of the first week (T1), at the end of the second week (T2), and at the end (‘post’) of the three-week stimulation session: the Fugl–Meyer Assessment for Upper Extremity (FMAUE), an index to assess the sensorimotor impairment; the Katz Index of Independence in Activities of Daily Living (KADL) for the independence in activities of daily living (ADL); the Timed Up and Go Test (TUG) to measure mobility [26]; and the Modified Ashworth Scale (MAS) [27] and Tardieu Scale (TaS) [28] for spasticity.

The Fugl–Meyer Assessment for Upper Extremity (FMAUE) consists of 33 items with a maximum score of 66. Each item is scored on a 3-point scale of 0 points (unable to perform), 1 point (partially able to perform), and 2 points (fully able to perform). The FMAUE includes the following assessment categories: shoulder–elbow–forearm function, wrist function, hand function (subscore C), and coordination/speed [29]. The FMAUE generally has a very high inter-rater reliability, especially the subscale for upper extremity motor skills, which is used in this study [30,31,32]. This test also shows high sensitivity for patients with severe to moderate impairment [29].

The Katz Index of Independence in Activities of Daily Living (KADL) assesses the ability to perform ADLs independently in six functions, including bathing, dressing, toileting, transferring, continence, and feeding. Patients score ‘yes’ or ‘no’ for independence in each of the six functions. A score of 6 indicates full function, 4 indicates moderate impairment, and 2 or less indicates severe functional impairment [33]. The KADL has an excellent intra-rater reliability (intercorrelation coefficient, ICC= 1.000) and internal validity (ICC = 0.999) [34].

To measure mobility, the change in time for the Timed Up and Go Test (TUG) will be assessed [26]. The TUG measures the time taken for a patient to get up from an armchair, walk 3 meters, turn around, walk back, and sit down again [26]. This widely used and well-known test has very good inter- and intra-rater reliability (ICC = 0.99) [26].

Spasticity is assessed using the Modified Ashworth Scale (MAS) [27]. The MAS is a semi-quantitative measure ranging from 0 to 4 points that is used to assess the severity of spasticity. MAS scores for the shoulder, upper arm flexor wrist, and finger flexors/extensors will be evaluated for each patient. The psychometric criteria of the MAS are described very controversially in the literature, ranging from unsatisfactory to very good [35,36]. For this reason, the Tardieu scale will also be used to assess spasticity.

The Tardieu Scale (TaS) quantifies spasticity by assessing the response of the muscle to different stretch velocities and by determining the spasticity angle. A goniometer is used to measure R2 and R1. The patient is seated during the test. The stretch velocity of V1 and V3 is used to measure R2 and R1, respectively. The quality of the muscle response is also assessed using the stretch velocity of V3. The difference between R2 and R1 is used as a measure of the dynamic component of spasticity [28]. The test–retest reliability of the Tardieu scale is reported to be moderate to very good [37], with good inter-rater and very good intra-rater reliability [38].

All assessments will be recorded by therapists who are not involved in the study. Before the study starts, standardized training will be given to all those responsible for collecting the assessments. Side effects will also be recorded and compared between groups.

### 2.5. Randomization and Blinding

We will use a blocked randomisation procedure. Eligible patients will be blindly randomized into one of two groups, the rPMS group or the NMS group, using a randomization box with opaque sealed envelopes. An independent and impartial person will carry out the randomisation procedure (1:1 allocation ratio). The assessments are collected by neutral therapists who are not involved in the study. The person administering the stimulation is not involved in the motor testing. The therapists providing the rehabilitation therapy are also blinded to the patients’ affiliation. The data analyst is unaware of the patients’ allocation.

### 2.6. Treatment

#### 2.6.1. Repetitive Peripheral Magnetic Stimulation (rPMS)

For rPMS, a figure-of-eight coil of MAGSTIM Rapid² will be used with a maximum magnetic field strength of 0.5–3.5 Tesla.

The patient will be seated during the stimulation. Stimulation begins with the arm flexors, then the arm extensors and the hand flexors, and it ends with stimulation of the hand extensors. The coil will be positioned flat along the muscle belly to induce large amplitudes of electrical current [39]. Before starting the stimulation session, the passive range of motion (pROM) of the elbow and hand will be determined. The stimulation intensity will be adapted to each patient and will depend on two criteria. First, the stimulation must be painless. If a patient finds the stimulation painful, the stimulation intensity will be reduced in 5% increments until the patient tolerates the stimulation intensity. Second, the stimulation intensity must be at least half the pROM of the muscle contraction. The stimulation intensity will be started with an intensity at 20% of the maximum stimulator output and increased or decreased in 5% increments. Patients who cannot tolerate sufficient stimulation intensity to induce a half pROM muscle contraction will be excluded.

**Figure 1:**
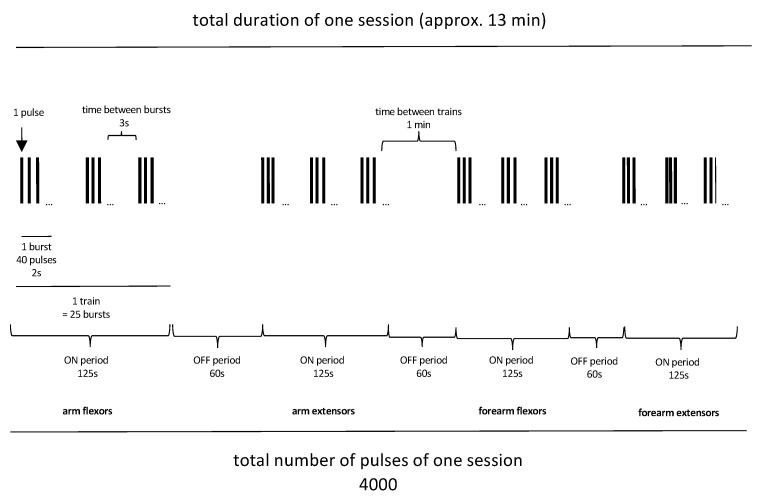
Stimulation protocol of rPMS.

The 20 Hz rPMS is applied in four trains. Each train consists of 25 bursts of 40 pulses. Each patient receives a total of 4000 pulses in a 13 min stimulation session (see Figure 1). Patients will receive one stimulation session per day, five days per week, from Monday to Friday, for three weeks, for a total of 15 stimulation sessions, with clinical assessment after the last stimulation day (post).

#### 2.6.2. Neuromuscular Stimulation (NMS)

In the NMS group, the electrical stimulation will be delivered using Wellcare Digi-Stim^®^ (Sanowell, Alzenau, Germany) with 4.0 × 4.0 cm^2^ electrodes placed on the skin. The patient will be seated during electrical stimulation. Electrical stimulation will start with the flexors and then the extensors of the arm and the hand. The same two criteria as in the rPMS group will be used to determine the stimulation intensity: a painless stimulation and a muscle contraction of at least 50% pROM. The stimulation frequency of the electrical stimulation will be 20 Hz, with an on phase of two seconds and an off phase of three seconds. Each muscle group will receive 25 muscle contractions in a 12 min session. Patients will be stimulated daily from Monday to Friday for three weeks, for a total of 15 stimulation sessions.

### 2.7. Outcome Measures

The primary outcome is the difference between the Fugl–Meyer Assessment for Upper Extremity (FMAUE_Diff_) scores before and after the three-week stimulation session, calculated as follows: FMAUE_Diff_ = (FMAUE_post_ − FMAUE_pre_)/FMAUE_pre_.

The secondary outcomes are KADL, TUG, MAS, and TaS, which are calculated in the same way as the primary outcome of FMAUE_Diff_.

Correlation analyses will be used to analyze the relationship between arm spasticity (MAS and TaS), arm function (FMAUE), and independence in ADL (KADL) in each intervention group (rPMS and NMS). For this reason, a sum score will be calculated for a total MAS score (MAS_total_) of all the muscles assessed. The change in this score (between pre and post, MAS_total-Diff_) and TaS (TaS_Diff_) is correlated with the change in FMAUE (FMAUE_Diff_) as well as with the change in KADL (KADL_Diff_).

For each patient, stimulation and testing will be performed at the same time from Monday to Friday between 09:00 and 12:00.

**Figure 2:**
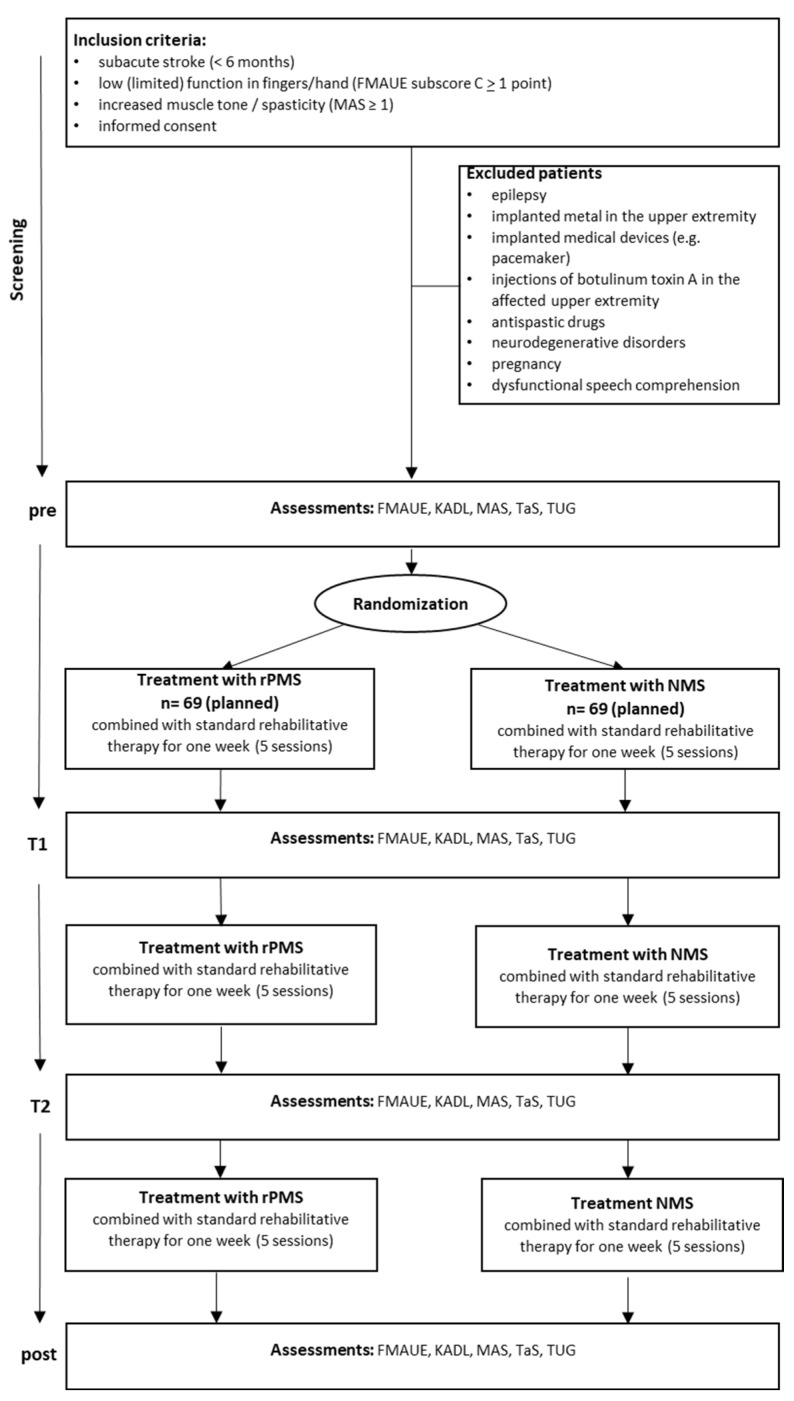
Study design. FMAUE = Fugl–Meyer Assessment for Upper Extremity; KADL = Katz Index of Independence in Activities of Daily Living (KADL); TUG = Timed Up and Go Test (TUG); MAS = Modified Ashworth Scale; TaS = Tardieu Scale.

During the trial, included patients may not be enrolled in other trials, treated with botulinum toxin or antispastic drugs, receive peripheral electrical stimulation, or receive peripheral or central magnetic stimulation (see Figure 2).

### 2.8. Statistical Analysis

Statistical analysis will be based on the intention to treat analysis using IBM SPSS Statistics 29 (IBM, Armonk, NY, USA). The Kolmogorov–Smirnov Test will be used to test for normal distribution. Levene’s test will be used to assess the equality of variances between groups. If there is no normal distribution, the Mann–Whitney-U test will be used; otherwise, analysis of variance (ANOVA) will be used to test for differences in the repeated measures of FMAUE (pre, T1, T2, and post). In this 2 × 2 factorial design, group will be the independent factor and time will be the dependent factor. Post hoc analyses will be performed for significant main effects and interactions. One-way ANOVA will be used to ensure that the baseline FMAUE scores before the test are not statistically significant between the two groups. The same analysis will also be performed for KADL, TUG, MAS, and TaS.

For the within-group analysis (pre- and post-test), a paired *t*-test will be used if the distribution is normal; otherwise, the Wilcoxon test will be used.

For FMAUE_Diff_, two-sample *t*-tests will be used to test for the difference between the two groups (rPMS and NMS) if the distribution is normal. If the distribution is not normal, the Wilcoxon test will be used. The same analysis will be used for KADL_Diff_, TUG_Diff_, MAS_Diff_, and TaS_Diff_.

To analyze the relationship between arm spasticity (MAS) and arm function (FMAUE) and independence in ADL (KADL) in each intervention group (rPMS and NMS), correlation analyses between the MAS_total-Diff_ and (TaS_Diff_) and FMAUE_Diff_ as well as KADL_Diff_ will be used.

For missing follow-up measurements, the Last Observation Carried Forward (LOCF) procedure will be applied by inserting the most recent present value before.

The level of statistical significance will be set at *p* < 0.05, and Bonferroni correction will be used for multiple comparisons.

The effect size will be calculated using Cohen’s *d* according to the formula mean difference between two groups divided by the result of the pooled standard deviation (the pooled standard deviation is calculated as (=√[(standard deviation rPMS^2^ + standard deviation NMS^2^)/2])). The effect size will be defined as small (0.2), medium (0.5), or large (0.8) [40].

The primary safety endpoint is the incidence of seizures and headaches during the intervention period.

### 2.9. Study Organization

Data Monitoring Controlling (DMC) will be performed by the Moritz Clinic. DMC is independent of patient recruitment.

The first patients of the pilot feasibility study to verify the power calculation were enrolled between October 2014 and May 2015.

### 2.10. Data Safety Monitoring

An independent Data and Safety Monitoring Board will monitor adverse events (e.g., seizures and headaches). Possible reasons for early termination of the entire study are a decision by the trial investigators in case of unacceptable risks based on the risk–benefit assessment and new scientific evidence during the trial. The decision to discontinue the study will be made by the principal investigator.

## 3. Perspective

REPMAST is an investigator-initiated, assessor-blinded, randomized controlled comparative interventional trial. The results of this study will be relevant to improving motor recovery after a stroke using peripheral stimulation. This approach has the potential to improve patients’ quality of life by promoting greater independence in performing activities of daily living (ADLs).

## Data Availability

All data will be made available upon request after the publication of the study results. The data are not available publicly due to being a part of an ongoing study.
